# Estimating correlations among demographic parameters in population models

**DOI:** 10.1002/ece3.5809

**Published:** 2019-11-21

**Authors:** Thomas V. Riecke, Benjamin S. Sedinger, Perry J. Williams, Alan G. Leach, James S. Sedinger

**Affiliations:** ^1^ Program in Ecology, Evolution, and Conservation Biology University of Nevada Reno Nevada; ^2^ Department of Natural Resources and Environmental Science University of Nevada Reno Nevada; ^3^ College of Natural Resources University of Wisconsin‐Stevens Point Stevens Point Wisconsin

**Keywords:** black brent, *Branta bernicla nigricans*, capture–recapture, demography, fitness, hyperpriors, inverse Wishart, multivariate normal

## Abstract

Estimating correlations among demographic parameters is critical to understanding population dynamics and life‐history evolution, where correlations among parameters can inform our understanding of life‐history trade‐offs, result in effective applied conservation actions, and shed light on evolutionary ecology. The most common approaches rely on the multivariate normal distribution, and its conjugate inverse Wishart prior distribution. However, the inverse Wishart prior for the covariance matrix of multivariate normal distributions has a strong influence on posterior distributions. As an alternative to the inverse Wishart distribution, we individually parameterize the covariance matrix of a multivariate normal distribution to accurately estimate variances (*σ* ^2^) of, and process correlations (*ρ*) between, demographic parameters. We evaluate this approach using simulated capture–mark–recapture data. We then use this method to examine process correlations between adult and juvenile survival of black brent geese marked on the Yukon–Kuskokwim River Delta, Alaska (1988–2014). Our parameterization consistently outperformed the conjugate inverse Wishart prior for simulated data, where the means of posterior distributions estimated using an inverse Wishart prior were substantially different from the values used to simulate the data. Brent adult and juvenile annual apparent survival rates were strongly positively correlated (*ρ* = 0.563, 95% CRI 0.181–0.823), suggesting that habitat conditions have significant effects on both adult and juvenile survival. We provide robust simulation tools, and our methods can readily be expanded for use in other capture–recapture or capture‐recovery frameworks. Further, our work reveals limits on the utility of these approaches when study duration or sample sizes are small.

## Graphical abstract

Specifying hyperpriors (blue) for components of covariance matrices, rather than inverse Wishart priors (red) allows for accurate estimation of process correlations of demographic components.

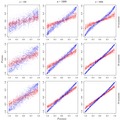

## 1 INTRODUCTION

Capture–mark–recapture–resight and capture–mark–recovery data can be used to estimate demographic parameters such as true and apparent survival, site fidelity, movement and harvest rates, breeding propensity, demographic heterogeneity, and relationships among these parameters and environmental covariates (Brownie & Pollock, 1985; Cam, Link, Cooch, Monnat, & Danchin, 2002; Gimenez, Cam, & Gaillard, 2018; Kendall et al., 2013; Kendall, Nichols, & Hines, 1997). Estimating relationships between demographic parameters can lead to more effective conservation actions (Arnold, Afton, Anteau, Koons, & Nicolai, 2016; Servanty et al., 2010, 2011), where biologists might direct conservation actions toward demographic components which are intrinsically linked, such as pre‐and postfledging survival (Nicolai & Sedinger, 2012) or adjust anthropogenic harvest rates to affect population growth rates of wild organisms (Nichols, Runge, Johnson, & Williams, 2007; Péron, 2013; Runge et al., 2002; Williams & Johnson, 1995). Estimating relationships among demographic rates can also advance our understanding of individual heterogeneity, life‐history trade‐offs, and the evolution of life histories (Cam, Aubry, & Authier, 2016; Cam et al., 2002; Gimenez et al., 2018; Stearns, 1992).

Estimating correlations among demographic parameters has often proven challenging, because sampling correlations can obscure process correlations (Anderson & Burnham, 1976; Link & Barker, 2005). For instance, the estimation of band recovery probability is confounded with the estimation of survival (Anderson & Burnham, 1976), affecting inference on the relationship between survival and harvest. Previous methods have employed approaches to achieve independent samples by comparing estimates of survival from the marked sample with estimates of harvest of the total population (Anderson & Burnham, 1976) or by partitioning the capture‐mark‐recovery data (Nichols & Hines, 1983). Recently, work on the effects of harvest on survival has focused on understanding process correlations (*ρ*) between survival and harvest rates (Arnold et al., 2016; Bartzen & Dufour, 2017; Sedinger, White, Espinosa, Partee, & Braun, 2010), where a strong negative correlation suggests additive relationships between survival and harvest, and minimal or no correlation may be indicative of compensation or partial compensation, although these relationships are complex (Arnold et al., 2016; Péron, 2013). Others have focused on the correlation between natural mortality and harvest mortality (Péron, 2013; Servanty et al., 2010), where no correlation may be indicative of additive harvest, and a negative correlation may be indicative of compensatory harvest. Ecologists have both highlighted (Arnold et al., 2016; Péron, 2013; Servanty et al., 2010) and debated (Arnold, Afton, Anteau, Koons, & Nicolai, 2017; Lindberg, Boomer, Schmutz, & Walker, 2017) the utility of these approaches. Capture–recapture (Riecke, Leach, Gibson, & Sedinger, 2018) and integrated population models can also be used to examine covariation among parameters such as survival and breeding propensity. This allows researchers to examine life‐history trade‐offs at the individual or population level (Cam et al., 2002), as well as correlations among other demographic parameters (Kindsvater et al., 2018; Koons, Arnold, & Schaub, 2017; Link & Barker, 2005; Schaub, Jakober, & Stauber, 2013). Conservation biologists can subsequently use these relationships to predict the potential of management actions to affect wildlife populations.

Bayesian hierarchical models allow for the separation of sampling and process correlations (Link & Barker, 2005). However, the choice of priors can dramatically impact posterior distributions and inference when using Bayesian models, where relatively uninformative priors are often favored (Link, Cam, Nichols, & Cooch, 2002) for objective Bayesian analyses. Further, conjugate priors are desirable computationally, because their full‐conditional distributions are analytically tractable with a known distribution, and there is no need for Metropolis–Hastings updates or tuning. A conjugate prior for the covariance matrix (*K* × *K*) of a multivariate normal distribution is the inverse Wishart distribution, where quantitative ecologists often use this distribution with degrees of freedom equal to *K* + 1 and a *K* × *K* identity matrix as a scale matrix (Haff, 1979; Kéry & Schaub, 2012; Péron, 2013; Wishart, 1928). Thus, for a bi‐variate normal distribution,

y∼Normal(μ,Σ)

(1) $$\Sigma =\left[\begin{array}{cc} \sigma_{1}^{2} & \sigma_{1}\sigma_{2}\rho_{1,2}\\ \sigma_{1}\sigma_{2}\rho_{1,2} & \sigma_{2}^{2} \end{array}\right],$$

$$\Sigma^{-1}∼\text{Wishart}\;\left(3,\;I\right),$$

where ***I*** is a 2 × 2 identity matrix. Critically, simulation work has revealed that the inverse Wishart prior is strongly informative for covariance matrices (Alvarez, Niemi, & Simpson, 2014). Specifically, the inverse Wishart prior can result in strong prior influence on the posterior distribution of variances and covariances, particularly when variances are small (*σ* ^2^ < 0.25), affecting the posterior distribution of correlations (Alvarez et al., 2014). The shape of the logistic link function, and life‐history theory (Stearns, 1992), dictates that the standard deviations (*σ*), and consequently variances (*σ* ^2^), of demographic parameters of interest will typically be small (Gimenez et al., 2018). For instance, typical North American dabbling duck survival rates exhibit variances of approximately *σ* ^2^ = 0.1, where *σ* ^2^ is from Equation (1). Given the structure of covariance matrices, where the covariance is equal to the product of the square roots of the applicable variances, and a process correlation between the applicable parameters (*σ_i_σ_j_ρ_i_*_,_ *_j_*), the use of the Wishart, scaled inverse Wishart (O'Malley & Zaslavsky, 2008), or hierarchical half‐t (Huang & Wand, 2013) priors for a precision matrix often leads to the underestimation of *ρ*. This has important implications for the interpretation of process correlations, affecting biological inference and management decisions.

To alleviate current issues regarding the estimation of ***ρ*** and ***σ*** ^2^ when using the logistic link and the inverse Wishart distribution, we propose individual parameterization for the components of the covariance matrix when using multivariate normal distributions to estimate parameters.

y∼Normal (μ,Σ)

(2) $$\Sigma =\left[\begin{array}{cc} \sigma_{1}^{2} & \sigma_{1}\sigma_{2}\rho_{1,2}\\ \sigma_{1}\sigma_{2}\rho_{1,2} & \sigma_{2}^{2} \end{array}\right],$$

$$\sigma_{i}∼\text{Uniform}\;\left(0,\;5\right),$$

$$\rho_{i,j}∼\text{Uniform}\;\left(-1,\;1\right),$$

**Figure 1:**
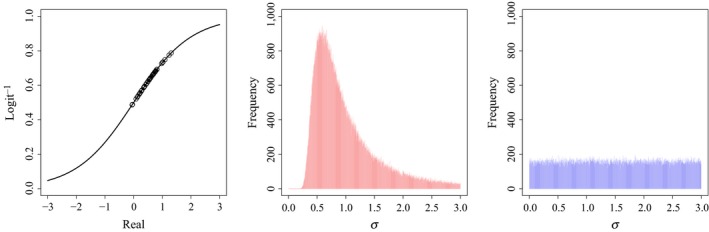
Fifty randomly generated annual survival probabilities given mean survival (*μ_S_*) of 0.5 and a variance ($\sigma_{S}^{2}$) of 0.1 on the real scale and their respective positions on the inverse logit link function (left). Additionally, we plot prior distributions for the standard deviation of survival probabilities from a Wishart prior with an identity scale matrix and degrees of freedom equal to *K* + 1 (center), and from a uniform prior with a lower bound of 0, and an upper bound of 5 (right), where the uniform prior is clearly less informative for variances of demographic parameters

This approach avoids introducing strong, undesirable effects on the posterior distributions via the prior distributions, such as the scaled inverse chi‐square distribution implied for the variances when using the inverse Wishart distribution as a prior for the covariance matrix (Figure 1), or the implied correlations among *σ* and *ρ* (Alvarez et al., 2014, Figure 2). The inverse Wishart prior, as well as other derivatives of that prior, clearly leads to correlations among *σ* and *ρ* (Alvarez et al., 2014). Other hyperpriors for variances, such as the half‐t, half‐normal, or half‐Cauchy, may be equally effective as a uniform prior. However, we strongly suggest careful consideration of the data, and the use of vague priors when modeling a covariance matrix.

**Figure 2:**
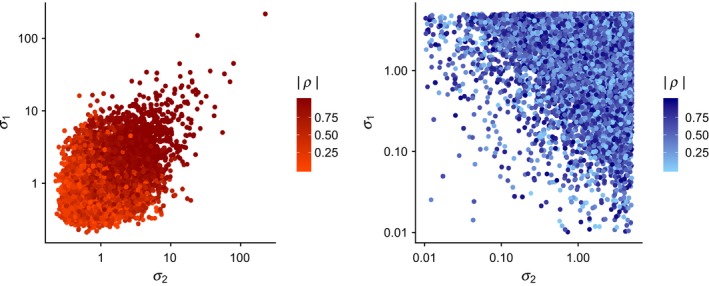
Correlations among standard deviations (*σ_k_*) and process correlations (*ρ*) drawn from an inverse Wishart prior with an identity scale matrix and degrees of freedom equal to *K* + 1 (left), and from a multivariate normal distribution where hyperpriors of the covariance matrix were individually specified, where we used a half‐normal distribution with a variance of ten for the standard deviations, and a uniform prior with lower and upper bounds of −1 and 1 for the process correlation (right). Note the strong correlation between values of (*σ_k_*) and values of (*ρ*) when using the inverse Wishart prior

To demonstrate the utility of our approach, we use simulated data, as well as a robust longitudinal dataset, capture–mark–recapture–resight data from female Pacific black brent geese (*Branta bernicla nigricans*; hereafter, brent) marked on the Yukon–Kuskokwim River Delta in Alaska (1988–2014). Brent breeding at the Tutakoke River Colony on the Yukon–Kuskokwim River Delta, Alaska, has been monitored from 1984 through the present (Leach et al., 2019; Sedinger, Flint, & Lindberg, 1995). Over the duration of the Tutakoke River Colony brent demographic project, researchers have collected data on every avian demographic component, where an enhanced understanding of process correlations among demographic components may lead to more effective management actions, and improve our understanding of demographic buffering and life‐history trade‐offs. We use multivariate normal distributions, and novel covariance matrix parameterizations for demographic parameters (Barnard, McCulloch, & Meng, 2000), to assess the relationships among adult and juvenile survival of brent to better understand life‐history trade‐offs and the demographic buffering hypothesis (Gaillard, Festa‐Bianchet, Yoccoz, Loison, & Toigo, 2000; Pfister, 1998; Stearns, 1992), which predicts that vital rates with greater demographic sensitivities will have lower variances. Further, we simulate thousands of biologically plausible capture–mark–recapture datasets to explore the effects of prior influence on inference in the presence of parameter uncertainty and imperfect observation when truth is known. Our work has important implications for researchers using multivariate normal distributions to estimate process correlations among demographic parameters of wild organisms, as well as research in other fields where inverse Wishart distributions are used as prior for covariance matrices for multivariate data (e.g., Multivariate spatial models, Gelfand, Diggle, Guttorp, & Fuentes, 2010; Bayesian structural equation models).

## 2 METHODS

### 2.1 Capture–mark–recapture data simulation

We simulated capture–mark–recapture datasets using mean annual adult apparent survival probabilities of 0.73 ($\mu_{\phi_{\mathrm{ad}}}=1,\sigma_{\phi_{\mathrm{ad}}}^{2}=0.1$ on the real scale), and mean annual juvenile survival probabilities of 0.27 ($\mu_{\phi_{\mathrm{juv}}}=-1,\sigma_{\phi_{\mathrm{juv}}}^{2}=0.1$ on the real scale; Table 1), to approximate adult and juvenile survival probabilities for species with intermediate‐paced life‐history strategies. Our simulations and the R script we provide (Data availability statement) can be easily modified to assess relationships for species with slower or faster paced life‐history strategies. To assess our ability to estimate process correlations (*ρ*), we used fixed variances of 0.1 for adult and juvenile survival rates, and randomly generated a process correlation between adult and juvenile survival for each simulation (*v*) using a uniform distribution to represent all possible relationships between these two demographic parameters,

$$\rho_{v}∼\text{Uniform}\;(-1,1),\;\text{for}\;v=1,\ldots ,V,$$

$$\sigma_{\phi_{\mathrm{ad}}}^{2}=\sigma_{\phi_{\mathrm{juv}}}^{2}=0.1,$$

(3) $$\text{logit}\;(\Phi )∼\text{Normal}\;(\mu_{\phi },\Sigma ),$$

$$\Sigma =\left(\begin{array}{cc} \sigma_{\phi_{\mathrm{ad}}}^{2} & \sigma_{\phi_{\mathrm{ad}}}\sigma_{\phi_{\mathrm{juv}}}\rho \\ \sigma_{\phi_{\mathrm{ad}}}\sigma_{\phi_{\mathrm{juv}}}\rho & \sigma_{\phi_{\mathrm{juv}}}^{2} \end{array}\right)$$

**Table 1:** Parameter values used to simulate data for capture–mark–recapture models

Model	Symbol	Parameter value
Parameter	*θ*	
Mean adult survival	$\mu_{\phi_{\mathrm{ad}}}$	1
Temporal variance in adult survival	$\sigma_{\phi_{\mathrm{ad}}}^{2}$	0.1
Mean juvenile survival	$\mu_{\phi_{\mathrm{juv}}}$	−0.5
Temporal variance in juvenile survival	$\mu_{\phi_{\mathrm{juv}}}^{2}$	0.1
Process correlation	$\rho_{\phi_{ad,}\phi_{\mathrm{juv}}}$	Uniform (−1, 1)
Detection probability	*p*	0.5

To generate capture–mark–recapture data, we first simulated each individual's latent state (*z_i_*_,_ *_t_*) given its intial release occasion (*τ_i_*), initial age‐class (juvenile or adult), number of release occasions (*T*), and time‐varying survival probabilities (Φ),

(4) $$z_{i,t}∼\left\{\begin{array}{cc} \text{Bernoulli (}\phi_{i,t}), & z_{i,t-1}=1\\ 0, & z_{i,t-1}=0 \end{array}\right.,\;\text{for}\;t=\tau_{i},\ldots ,T.$$

We then simulated capture histories for each individual (*y_i_*_,_ *_t_*) given its current latent state (*z_i_*_,_ *_t_*) and a detection probability (*p* = .5),

(5) $$y_{i,t}∼\left\{\begin{array}{cc} \text{Bernoulli (}p), & z_{i,t}=1\\ 0, & z_{i,t}=0 \end{array}\right.,\;\text{for}\;t=\tau_{i}+1,\ldots ,T.$$

We simulated 1,000 populations and capture–mark–recapture datasets for each pairwise comparison of 10, 20, and 30 occasions, and 100, 1,000, and 5,000 releases per occasion, for a total of 9,000 simulated capture–mark–recapture datasets.

### 2.2 Analyzing the simulated data

For each simulated dataset, we built age‐specific Bayesian CJS models (Cormack, 1964; Jolly, 1965; Seber, 1965), identical to the model used to simulate the data. We first modeled each individual's latent state (*z_i_*_,_ *_t_*) given its release occasion (*τ_i_*) and age at release, given Equation (4). We then modeled each individual's capture history as a function of its latent state, given Equation (5). For computational efficiency, we structured the capture–mark–recapture data in *m* arrays for juveniles and adults (Data availability statement). To examine process correlations between adult (*ϕ_ad_*) and juvenile (*ϕ_ad_*) survival, we drew occasion specific adult and juvenile survival probabilities from a multivariate normal distribution,

(6) logit(Φ)∼Normal(μ,Σ),

where $\mu =(\mu_{\phi_{\mathrm{ad}}},\mu_{\phi_{\mathrm{juv}}})$ and $\Phi =(\phi_{ad,t},\phi_{juv,t})$. We specified vague priors for the means of adult and juvenile survival,

(7) $$\begin{array}{c} \mu_{\phi_{\mathrm{ad}}}∼\text{Normal}\;\left(0,1\right),\\ \mu_{\phi_{\mathrm{juv}}}∼\text{Normal}\;\left(0,1\right). \end{array}$$

We then used two approaches to estimate the covariance matrix (**Σ**). First, we modeled the precision matrix (**Σ** ^−1^) of the multivariate normal distribution with a Wishart prior (Equation 1). Second, we specified priors for the components of the covariance matrix (Equation 2).

### 2.3 Brent capture–mark–recapture data collection

We captured brent by drive‐trapping during brood‐rearing and the adult remigial molt at the Tutakoke River Brent Colony in western Alaska (61.25°N, −165.62°W; Sedinger, Lindberg, Rexstad, Chelgren, & Ward, 1997). We uniquely marked all encountered individuals with stainless steel or incoloy USGS rings and uniquely engraved plastic tarsal rings. Prior to ringing efforts each year, we monitored nests in 49 long‐term, 50‐m radius plots, and actively located nests of tarsal‐ringed females at the colony (Sedinger et al., 1995). Following hatch, we observed adults and goslings from 3 to 4 m tall observation blinds throughout brood‐rearing. We then captured adults, goslings, and second‐year females (fledged the previous year) during the adult and second‐year remigial molt, and prior to goslings gaining flight capabilities, with few exceptions. For this analysis, we constrained our encounter histories to include all adult (*ad*), second‐year (*sy*), and juvenile (*juv*) females encountered at the nest, during brood‐rearing, or during ringing drives from 1988 to 2014, for a total sample of 8,338 adult and second‐year, and 12,630 juvenile, female brent.

### 2.4 Brent capture–mark–recapture data analysis

We built models to estimate adult and juvenile survival, and the correlation between these parameters, in exactly the same way as for the simulated data with one exception. We modeled age‐related and temporal heterogeneity in detection probability. Brent do not breed until after their second year, and they are less likely to be detected at the breeding colony until this time. However, we do resight second‐year females at the colony and recapture these individuals during ringing efforts. Thus, we modeled detection probabilities of second‐year and adult brent separately based on age‐class (*k*), where we estimated detection probability for both age‐classes (*p_k_*) as random variables drawn from a normal distribution with a mean ($\mu_{p_{k}}$) and variance ($\sigma_{p_{k}}^{2}$),

(8) $$\begin{array}{c} \text{logit}\left(p_{k,t}\right)=\beta_{0}+\epsilon_{p_{k,t}},\\ \beta_{0}∼\text{Normal}\;\left(0,3\right),\\ \epsilon_{p_{k,t}}∼\text{Normal}\;\left(0,\sigma_{p_{k}}^{2}\right),\\ \sigma_{p_{k}}∼\text{Uniform}\;\left(0,3\right). \end{array}$$

We did not discriminate between adult and second‐year survival probabilities, as previous analyses have not indicated variation in survival rates between these age‐classes (Leach et al., 2019; Lindberg, Sedinger, & Lebreton, 2013; Sedinger, Herzog, & Ward, 2004). To examine process correlations between adult (*ϕ_ad_*_,_ *_t_*) and juvenile (*ϕ_juv_*_,_ *_t_*) survival, we drew survival parameters (Φ) from a multivariate normal distribution. We used identical approaches as those used for the simulated data to parameterize the covariance matrix, where we first modeled the precision matrix (**Σ** ^−1^) of the multivariate normal distribution with a Wishart prior (Equation 1). Second, we specified hyperpriors for the components of the covariance matrix (Equation 2).

### 2.5 Computational details

We simulated data using R 3.5.1 (R Core Team, 2018), and all analyses were conducted in JAGS (Plummer, 2003) using the ‘jagsUI’ package (Kellner, 2016). We ran two MCMC chains of 25,000 iterations for each model for simulated data, where we discarded the first 15,000 iterations and retained every fifth saved iteration (Schaub & Fletcher, 2015). For the brent data, we ran two MCMC chains of 100,000 iterations, where we discarded the first 50,000 iterations and retained every fifth saved iteration.

## 3 RESULTS

### 3.1 Capture–mark–recapture simulation

**Figure 3:**
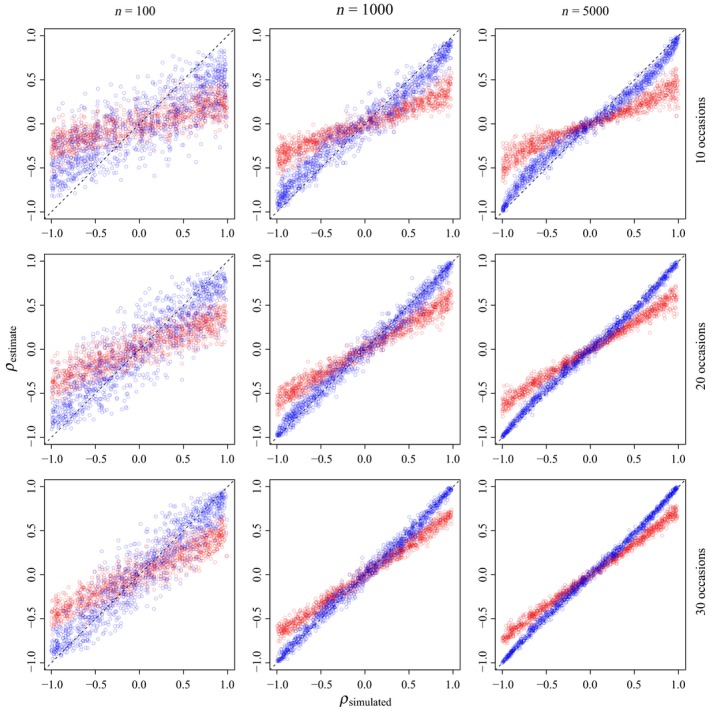
Means of the posterior distributions for process correlations (*ρ*) among adult (*ϕ_ad_*) and juvenile (*ϕ_juv_*) survival estimated from simulated capture–mark–recapture data using inverse Wishart (red) and hyperprior (blue) parameterizations for the covariance matrix of the multivariate normal distribution from which the parameters were estimated for different combinations of releases (*n* = 100, 1,000, or 5,000) and occasions (*t* = 10, 20, or 30). The dashed line represents 1:1 congruence between the simulated data and the estimate

When we used the same model to simulate and analyze the capture–mark–recapture data with an inverse Wishart prior on the covariance matrix of the multivariate normal distribution, the means of the posterior distributions of the process correlation (*ρ*) between adult and juvenile survival were different than the values used to simulate the data for all combinations of releases and occasions (Table 2). Further, 95% Bayesian credible intervals only covered truth for approximately 75% of simulations (Table 3). Critically, coverage declined as |*ρ*| increased (Figure 3). However, when we used hyperpriors on the individual components of the covariance matrix, matching the variances of the demographic parameters to the prior (Figure 1), bias in the estimation of *ρ* was reduced for all combinations of releases and occasions (Figure 3), and eliminated when data were sufficient to estimate these parameters (Table 2). Further, credible interval coverage was adequate for all combinations of releases and occasions (Table 3).

**Table 2:** Mean absolute value of the difference between the simulated process correlation used to generate the data, and the mean of the posterior distributions of the estimated process correlations (*ρ*), as a function of the number of occasions (*t*), the number of individuals released per occasion (*n*), and the parameterization of the covariance matrix used for the capture–recapture models

Parameterization	Occasions		
Releases	10	20	30
Inverse Wishart			
100	0.426	0.346	0.325
1,000	0.329	0.227	0.197
5,000	0.280	0.163	0.147
Hyperpriors			
100	0.319	0.126	0.095
1,000	0.203	0.067	0.042
5,000	0.150	0.048	0.029

**Table 3:** The proportion of simulations (out of 1,000) for which the value of *ρ* used to simulate the capture–mark–recapture data was included in the 95% Bayesian credible interval of the estimate of *ρ* as a function of the number of occasions (*t*), the number of individuals released per occasion (*n*), and the parameterization of the covariance matrix used for the capture–recapture models

Parameterization	Occasions		
Releases	10	20	30
Inverse Wishart			
100	0.654	0.696	0.720
1,000	0.645	0.675	0.737
5,000	0.617	0.744	0.743
Hyperpriors			
100	0.966	0.996	0.999
1,000	0.986	0.999	1.000
5,000	0.986	0.997	0.996

### 3.2 Correlations among survival rates of female brent

**Figure 4:**
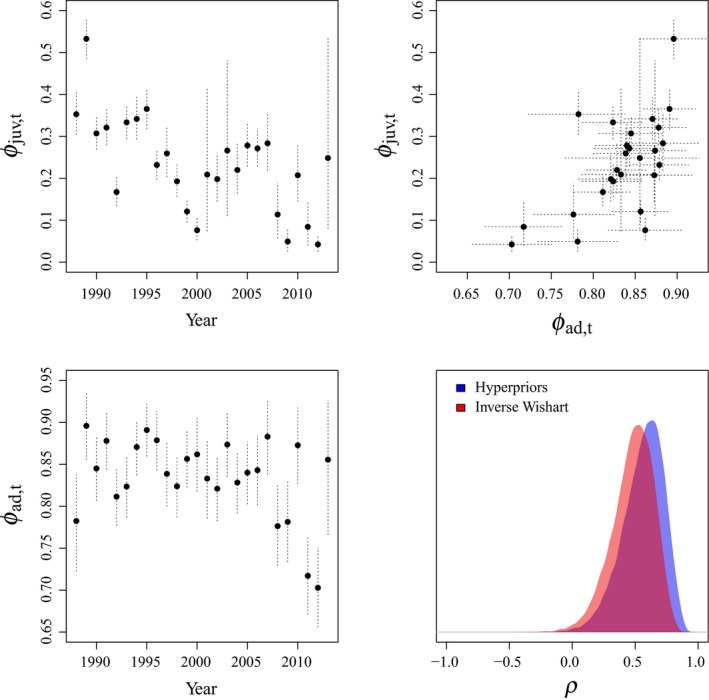
Means and 95% Bayesian credible intervals of adult (top left) and juvenile (bottom left) survival for black brent (*Branta bernicla nigricans*) marked on the Yukon–Kuskokwim River Delta, Alaska, 1988–2014. Further, we plot the correlation between these two parameters (top right), and the posterior distributions of the correlation (*ρ*) between these parameters from models using either an inverse Wishart prior (red), or hyperpriors (blue) for the covariance matrix components (bottom right)

**Figure 5:**
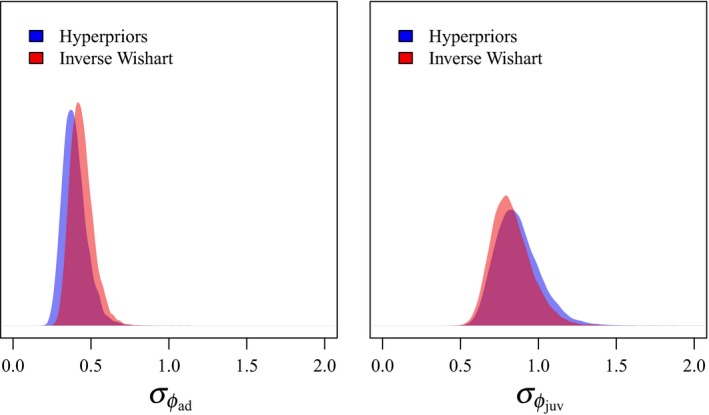
Density plots of the posterior distributions of variances (*σ* ^2^) of adult (left) and juvenile (right) survival for black brent (*Branta bernicla nigricans*) marked on the Yukon–Kuskokwim River Delta, Alaska, 1988–2014 from models with inverse Wishart priors on the covariance matrix (red), as well as models with hyperpriors on the individual components of the covariance matrix (blue)

When we used an inverse Wishart distribution as a prior for the covariance matrix, adult and juvenile survival of female brent were positively correlated (*ρ *= 0.481, 95% CRI 0.115–0.754; Figure 4), and the variance of juvenile survival ($\sigma_{\phi_{\mathrm{juv}}}^{2}=0.685$, 95% CRI 0.380–1.251) was significantly greater than the variance of adult survival ($\sigma_{\phi_{\mathrm{ad}}}^{2}=0.194$, 95% CRI 0.103–0.369). When we used hyperpriors for components of the covariance matrix, which more accurately reflected the potential variance of demographic components in our simulations, we estimated a stronger correlation between adult and juvenile survival (*ρ* = 0.563, 95% CRI 0.181–0.823; Figure 4). Further, estimates of the variances of juvenile survival ($\sigma_{\phi_{\mathrm{juv}}}^{2}=0.753$, 95% CRI 0.405–1.413) and adult survival ($\sigma_{\phi_{\mathrm{ad}}}^{2}=0.156$, 95% CRI 0.073–0.326) also differed from the inverse Wishart parameterization (Figure 5), where the difference between the variances increased.

## 4 DISCUSSION

### 4.1 Implications for the estimation of process correlations

Our simulation results highlight two critical issues in the estimation of process correlations among demographic components. First, we demonstrate the influence of currently used prior distributions on biological inference due to the informative nature of the inverse Wishart prior for the variances and covariances of demographic parameters (Figure 1). The Wish^−1^ (3, I_2×2_) prior implies certain hyperpriors for the variances of, and correlations between, demographic parameters which strongly impact inference. We also illustrate the inherent link between the estimation of *ρ* and *σ* when using inverse Wishart priors, where there is an intrinsic link between prior values for variances and process correlations (Figure 2). Our simulations clearly reveal that these issues can be addressed by specifying hyperpriors for the individual components of a covariance matrix. Second, and critically, we show that analyses with few releases (*n* < 100) or of short duration (*t* < 20), may fail to accurately estimate the underlying process correlation among demographic components (Figure 3), even when appropriate priors are used. These approaches have important utility for examining relationships between survival and harvest. Further, they allow researchers to examine life‐history trade‐offs among demographic components at both the population and individual level. Quantitative ecologists can use similar approaches to examine individual covariation in life‐history traits, such as survival and breeding probability (Cam et al., 2002), trade‐offs between current and future reproductive allocation (Leach et al., 2019), or correlations among demographic parameters and abundance or environmental conditions.

### 4.2 Implications for black brent populations

The estimated variance of adult survival rates was significantly less than the variance of juvenile survival rates (Figure 5). This is consistent with the demographic buffering hypothesis, where we would expect population growth rates of long‐lived organisms to be most sensitive to adult survival (Gaillard et al., 2000; Pfister, 1998). Therefore, adult survival should be the most consistent demographic component and be buffered from environmental variation or reproductive allocation (Gaillard et al., 2000; Pfister, 1998). As predicted, and indicated by previous research (Leach et al., 2017), we observed positive correlations between adult and juvenile survival. Brent family groups remain together from hatch through late spring (Sedinger, Nicolai, Lensink, Wentworth, & Conant, 2007), where we would expect environmental variation which impacts adult survival to affect juvenile survival as well, despite strong carry‐over effects of environmental conditions during growth on first‐year survival, and lifetime fitness, of juvenile brent (Riecke et al., 2018; Sedinger et al., 1995, 2007). Long‐term declines in both adult (Leach et al., 2017; Riecke et al., 2018) and juvenile survival (Leach et al., 2017) have critical implications for brent populations, which are also experiencing declines in fecundity (Ward, Amundson, Stehn, & Dau, 2018) and population size (Sedinger, Riecke, Leach, & Ward, 2019).

### 4.3 Implications for future research

We examine the behavior of multivariate normal distributions for demographic parameters in the presence of parameter uncertainty for a variety of biologically plausible scenarios. We suggest that investigators simulate data and conduct power analyses prior to drawing inference from their data. We provide simple R script to perform these analyses, where short‐term (<10 years) datasets with small sample sizes (<100 releases per year) generally yield inaccurate estimates of process correlations when using capture–mark–recapture or capture‐mark‐recovery data. Our work has important implications for previous (Péron, 2013; Servanty et al., 2010) and future research, and our parameterizations can be expanded to covariance matrices of *K* × *K* dimensions (Alvarez et al., 2014). Thus, our preliminary analyses highlight the power and importance of robust longitudinal datasets and the importance of model parameterization. Additionally, our simulations do not induce variation or heterogeneity in detection probability (*p*), where variation in these probabilities can affect the absolute bias and precision of estimates of *ρ* (e.g., increased detection probabilities or recovery and reporting rates lead to increased precision and accuracy in the estimation of *ρ*).

These approaches have great utility for the future development of integrated population and capture–mark–recapture‐recovery models, where quantitative ecologists can draw demographic parameters of interest from multivariate normal distributions within integrated population or capture–mark–recapture models to share information among demographic parameters. Finally, recent research (Alvarez et al., 2014) has revealed that the scaled inverse Wishart distribution (O'Malley & Zaslavsky, 2008) and hierarchical half‐t prior distribution proposed by Huang and Wand (2013) are less informative than the inverse Wishart, although they experience similar issues, albeit at a lesser scale (Alvarez et al., 2014). We do not believe we have completely resolved this issue, and recent advances in Bayesian methods (Gelman, Lee, & Guo, 2015) may further our understanding. Finally, our simulations indicate that specifying hyperpriors for the components of a covariance matrix is significantly more effective than using the inverse Wishart distribution, or other recently developed approaches (Huang & Wand, 2013; O'Malley & Zaslavsky, 2008) for capture–mark–recapture and other demographic analyses, as the hyperprior approach leads to accurate estimates of process correlations and variances given sufficient sample sizes. These results have important implications for a variety of multivariate models in ecology.

## CONFLICT OF INTEREST

None declared.

## AUTHOR CONTRIBUTIONS

J.S.S. conceived the capture‐mark‐resight‐recapture study and has led data collection efforts through the course of the project. T.V.R., J.S.S., B.S.S., and A.G.L. collected data. T.V.R. and B.S.S. identified issues with existing parameterizations of these model types, with important contributions from all authors. T.V.R. designed the simulation experiment, with important contributions from P.J.W., T.V.R., and J.S.S. led the writing of the manuscript. All authors contributed critically to manuscript revisions and gave final approval for publication.

## Data Availability

R script for simulating capture–mark–recapture data and analyzing the data in JAGS, and RDATA and R script for analyzing the brent capture‐mark‐resight‐recapture data, are archived at the Dryad Digital Repository (https://doi.org/10.5061/dryad.dbrv15dws) https://datadryad.org/stash/share/jA19ZtMLZpLmIPIJDAGKFgjJSVvALvfJQWIqWnIfH0E.
